# A structural equation modelling of the direct and indirect factors associated with functional status over time as measured by WHODAS-32 items among postpartum women in Northwest Ethiopia

**DOI:** 10.1186/s13690-023-01055-w

**Published:** 2023-03-18

**Authors:** Marelign Tilahun Malaju

**Affiliations:** Department of Public Health, College of Health Sciences, Debre Tabor University, Debre Tabor, Ethiopia

**Keywords:** Structural equation modelling, Functional status, Direct and indirect factors, Postpartum women, WHODAS-32 items

## Abstract

**Introduction:**

Full functional status recovery which is a multidimensional concept, that includes personal care, infant and family care, social and occupational activities and adjusting to the role of motherhood may require several months to achieve. However, most study designs on postpartum maternal functional status were cross-sectional, providing limited insights into functional status patterns over time and the mediating relationships among variables associated with it during the postpartum period.

**Objective:**

To investigate the patterns of functional status over time and the mediating relationships among variables associated with functional status after childbirth.

**Methods:**

From October 2020 to March 2021, 775 women in Northwest Ethiopia participated in a community-based follow-up study that was linked to a health institution. Functional status was measured by the Amharic version of WHODAS 2.0 instrument. Structural equation modelling was used to determine the direct and indirect effects of predictor variables on individual domains of functional status as measured by WHODAS 2.0 instrument.

**Results:**

Higher fear of childbirth score, anxiety and PTSD score had a direct deleterious effect (increased risk of functional disability) on the overall functional status and six domains of WHODAS 2.0 (cognition, mobility, self-care, getting along with people, household life activities and community participation) at the first, second and third follow up periods. Higher social support had a direct protective effect (decreased risk of functional disability) on all domains of WHODAS 2.0 and the overall functional status at the three follow up periods. Higher social support had also an indirect protective effect through fear of birth on the six domains of WHODAS 2.0 and the overall functional status throughout the follow up period. Higher PTSD symptom score had also an indirect deleterious effect (increased risk of functional disability) through fear of birth on the overall functional status and six domains of WHODAS 2.0 (higher disability) throughout the follow up period. Complications of delivery management had a direct deleterious effect (increased disability score) on the domains of getting along with people, household life activities, mobility, self-care and community participation and on the overall functional status disability score.

**Conclusion:**

Maternal functioning in the postpartum period is initially impaired, but improves over time. Despite improvement, maternal morbidities are correlated with worse functioning scores compared to women without these morbidities. Interventions should target on the mediating role of fear of child birth, life threatening event of health risk and PTSD with the deleterious effects of complications of delivery management, poor social support, vaginal mode of delivery, anxiety, poor physical and mental quality of life on functional status of postpartum women.

**Supplementary Information:**

The online version contains supplementary material available at 10.1186/s13690-023-01055-w.

## Introduction

The postpartum period is a time commences one hour after the birth of a child and lasts up to six weeks after delivery, during which the mother’s body returns to a non-pregnant state [1–3]. It is also considered as the transitional period for the mother, newborn and the family as a whole [1–4]. This period is described as a process whereby there may be a lot of physical, emotional and social changes encountered by the women resulting in limitations in the performance of daily activities and impacting the functioning of women [4–7]. 

Postnatal care, which has an important place within the mother and child health services, is a preventive health service [2, 3]. According to the World health organization (WHO), postnatal care service is a constellation of preventive care, practices, and assessments designed to identify and manage maternal and newborn complications during the first six weeks after birth [2, 3]. The WHO recommends 4 postpartum visits: within the first 24 h, on the third day, between days 7 and 14, and at six weeks after childbirth [2, 3]. The aim of postnatal care is to maintain and promote the physical and mental health of a mother [1, 8]. In addition, it is intended to assess the social and cultural issues that can affect the health and wellbeing of a woman, her baby, family and the community at large [1, 8]. Therefore, as one of the goals of postnatal care, evaluation of maternal functioning after childbirth is essential to measure the effectiveness of postnatal services [4, 9]. 

Literatures on the concept of functional status after childbirth has focused on the physiological return to full function after delivery and states that mothers recover after six weeks of delivery [4]. However, full functional status recovery which is a multidimensional concept, that includes personal care, infant and family care, social and occupational activities and adjusting to the role of motherhood may require several months to achieve [4, 10, 11]. In addition, previous researches on maternal functioning during the postpartum period were cross-sectional, and those longitudinal studies were limited at investigating only direct predictors, providing limited insights into the mediating relationships among variables associated with functional status during the postpartum period [4, 12–16]. To the best of the author’s knowledge, there is no study which has tried to explore the mediating relationships among variables associated with functional status over time in the postpartum women. Particularly, there is no research in Ethiopia which has investigated the mediating relationships among variables associated with functional status after childbirth.

In order to reduce this knowledge gap, the present study was conducted aiming to identify patterns of functional status over time among postpartum women and to assess the mediating relationships among variables associated with functional status using a structural equation modelling [17]. Understanding the direct and indirect association (mediating relationships) of predictor variables with functional impairment could enable us to improve maternal functional status by employing intervention strategies that could disrupt the causal pathways of these variables [18]. Therefore, the aim of this study was to investigate the patterns of functional status over time and the mediating relationships among variables associated with functional status after childbirth using a structural equation modelling among postpartum women in Northwest Ethiopia.

## Methods

### Study design and study area

Data from a prospective follow-up study which was conducted in Northwest Ethiopia was evaluated; details of the methodology were covered in earlier works [4, 19–21]. In this prospective study, postpartum mothers were recruited in four hospitals of South Gondar zone, Northwest Ethiopia. Between October 1, 2020, and March 30, 2021, the data were collected. 

### Sampling procedure

The study included all women who had been diagnosed with any maternal morbidities, whether direct or indirect [4]. Women without direct or indirect maternal morbidities were chosen with simple random sampling technique. Women were asked if they would be willing to participate in the study after giving birth and before being discharged. Women who accepted to participate in the study had their full address collected, and an appointment was set to collect the data for the follow-up study at their residence. 

### Outcome variable

The absolute score of functional status was considered as the outcome variable of the study.

### Independent variables

The independent variables that were considered to have a direct and indirect association with the functional status in this study were: direct maternal morbidities (obstetric hemorrhage, hypertensive disorders, obstructed labour, puerperal sepsis, gestational diabetes mellitus, perineal tear), indirect maternal morbidities (anemia, malaria, hypertension, asthma, tuberculosis, HIV), complications of delivery management (perineal tear, vaginal wall/perineal laceration, episiotomy and cesarean section wound infection), sociodemographic variables (age, educational status, marital status, religion, ethnicity, occupation, monthly expenditure), residence, obstetric variables (parity and mode of delivery) and psychosocial factors (social support, fear of childbirth, depression and anxiety).

### Sample size determination

Using Epi-Info software Version 7, the sample size was calculated by comparing two proportions [4]. Accordingly, a minimum sample size of 753 was calculated by taking level of α as 0.05, power of 90%, odds ratio(OR) of 1.96, proportion of 15.4% [4, 12], ratio of 1:2 and by adding 10% non-response rate[4, 12]. A proportional allocation of the total sample size was carried out to reach the necessary sample size in each hospital based on the number of giving birth moms who visited each hospital during the previous year (as reported in each hospital's annual report).

### Operational definition of functional disability

The WHODAS 2.0 instrument's 32-item form was used to measure maternal functional status (the 32-item form was used for participants who were unemployed and no longer in school) [4]. The WHODAS has been previously validated and used in Ethiopia [4, 16, 22–24]. The WHODAS 2.0 is intended to assess daily living activity functioning and involvement during the last 30 days [4]. The tool offers a standard method for determining how any health condition affects functioning. It can be used to compare disability caused by various disorders since it is not particular to any one disease [4]. Mobility, self-care, getting along with people, life activities, and participation in society are the six domains that make up the WHODAS 2.0. Results included an overall score as well as functioning within the domains. WHODAS 2.0 scores can range between 0 and 100, with higher scores indicating more impairment of daily functioning [4, 25].

### Tools used for measurement of variables

The abbreviated form of the Depression, Anxiety and Stress Scale-21 questionnaire was used to measure depression, anxiety, and stress [4, 26–28]. The Posttraumatic Stress Disorder Checklist for DSM-5 (PCL-5) comprising the 20 PTSD symptoms (criterion B, C, D and E) was used to measure PTSD over the past month. Fear of childbirth was assessed using the Wijma Delivery Expectation/Experience Questionnaire (W-DEQ) [29, 30]. The Oslo three-item Social Support Scale, which has scores ranging from 3 to 14, was used to measure social support [31–33]. All these instruments had a standard cutoff and the details of the instruments are provided in the online supplementary material 1. 

### Domestic violence

By using a multi-country WHO survey questionnaire from 2005, domestic violence was measured at the third follow up period (18^th^ week of postpartum period). This survey includes three questions about sexual violence, six questions about physical violence, and four questions about psychological violence [34].

### Data collection and quality control

Before the women were discharged from each hospital, baseline questionnaires were collected by medical staff members working in the gynecology and obstetrics ward. Community health professionals (health extension workers in Ethiopian context) collected the 32-item WHODAS 2.0 follow-up data [4]. Based on the home visit appointment that was scheduled at the time of discharge, the follow-up data collection was carried out at the 6^th^, 12^th^ and 18^th^ weeks of the postpartum period. Health care professionals in the gynecology and obstetrics ward of the study institutions who collected the baseline data and community health workers who collected the follow-up data both received training. Pretest was done and on the basis of the results of the pretest, the questionnaire was modified for word corrections. Supervision was done by the principal investigator.

### Data processing and analysis

Stata version 16 and IBM SPSS Statistics Version 26.0 were used for the statistical analyses. The direct and indirect effects of independent variables on the individual domains of functional status (as measured by WHODAS 2.0) were assessed using the linear structural equation modelling. A multivariate technique called structural equation modeling (SEM) combines elements of multiple regression and factor analysis to simultaneously estimate a number of dependent relationships. Multi-collinearity can therefore be modeled and evaluated in SEM [17]. The direct, indirect, and total effects of independent variables on functional status were reported in the form of standardized beta coefficients [21]. Estimated effects for which p < 0.05 were considered as being statistically significant. A confirmatory factor analysis (CFA) was carried out to evaluate the measurement model fitness of the WHODAS 2.0 before fitting the structural equation model (SEM) [21].

The model fitness was determined using the comparative fit index (CFI), Tucker-Lewis’s index (TLI), root-mean-square approximation error (RMSEA) with a Sartorra-Bentler correction method. The TLI and CFI should both be greater than 0.90, and the RMSEA value should be less than 0.08, in order to assess whether the model is reasonably fitting the data [21, 35, 36]. The details of model fitness criteria are provided in the online supplementary material 2. 

### Ethical considerations

Approval was obtained, informed consent performed (with guardian if appropriate), and data treated confidentially.

## Results

### Sociodemographic characteristics by direct and indirect maternal morbidity status

The sociodemographic characteristics of the study participants are pretended in Table 1. Almost all of them (99.9%) were Amhara by Ethnicity and the majority (95.7%) were followers of Orthodox religion. Out of the total number of women participated in the study, 32.5% and 27.1% of them were found to have direct and indirect maternal morbidities.

**Table 1:** Sociodemographic characteristics of women by direct and indirect maternal morbidity status in Northwest Ethiopia, 2021

Variables	Direct maternal morbidity		Indirect maternal morbidity		Total n (%)
	Yes. n (%)	No. n (%)	Yes. n (%)	No. n (%)	
**Age** [Mean(± SD) = 26.33(± 4.355)]					
**Residence**					
Urban	251 (32.4)	520 (67.1)	210 (27.1)	561 (72.4)	771 (99.5)
Rural	1 (0.1)	3 (0.4)	0 (0.0)	4 (0.5)	4 (0.5)
**Ethnicity**					
Amhara	252 (32.5)	522 (67.4)	210 (27.1)	564 (72.8)	774 (99.9)
Tigre	0 (0.0)	1 (0.1)	0 (0.0)	1 (0.1)	1 (0.1)
**Religion**					
Orthodox	241 (31.1)	501 (64.6)	199 (25.7)	543 (70.1)	742 (95.7)
Muslim	10 (1.3)	20 (2.6)	10 (1.3)	20 (2.6)	30 (3.9)
Protestant	1 (0.1)	2 (0.3)	1 (0.1)	2 (0.3)	3 (0.4)
**Education status**					
Illiterate/read and write	31 (4.0)	34 (4.4)	26 (3.4)	39 (5.0)	65 (8.4)
Grade 1–8	48 (6.2)	88 (11.4)	40 (5.2)	96 (12.4)	136 (17.5)
Grade 9–12	74 (9.5)	145 (18.7)	58 (7.5)	161 (20.8)	219 (28.3)
Certificate/Diploma	63 (8.1)	154 (19.9)	53 (6.8)	164 (21.2)	217 (28.0)
Degree and higher	36 (4.6)	102 (13.2)	33 (4.3)	105 (13.5)	138 (17.8)
**Occupation**					
Gov't employed	61 (7.9)	169 (21.8)	53 (6.8)	177 (22.8)	230 (29.7)
Merchant/Student	39 (5.0)	106 (13.7)	33 (4.3)	112 (14.5)	145 (18.7)
Housewife	141 (18.2)	226 (29.2)	117 (15.1)	250 (32.3)	367 (47.4)
Farmer/Daily laborer	11 (1.4)	22 (2.8)	7 (0.9)	26 (3.4)	33 (4.3)
**Marital Status**					
Married	246 (31.7)	516 (66.6)	205 (26.5)	561 (72.4)	762 (98.3)
Single/widowed/divorced	6 (0.8)	7 (0.9)	5 (0.6)	4 (0.5)	13 (1.7)
**Monthly expenditure**					
< = 3000 Ethiopian currency	48 (6.2)	158 (20.4)	64 (8.3)	142 (18.3)	206 (26.6)
3001–4000 Ethiopian currency	76 (9.8)	116 (15.0)	48 (6.2)	144 (18.6)	192 (24.8)
> = 4001 Ethiopian currency	128 (16.5)	249 (32.1)	98 (12.6)	279 (36.0)	377 (48.6)

### Results of maternal morbidities and psychosocial variables

Among the leading direct maternal morbidities, pregnancy related infections (20.1%) were the most common maternal morbidities, followed by complications of delivery management (18.1%). With regard to the indirect maternal morbidities, the frequently occurring morbidities were preexisting diseases before pregnancy (23.1%). Anemia due to iron deficiency or vitamin B12 and/or folate deficiency which were classified as other maternal diseases (21.8%) were the second common indirect maternal morbidities. As indicated in Table 2, of the mental health problems, anxiety symptoms were the most common (18.5%) followed by depression symptoms (15.5%).

**Table 2:** Results of maternal morbidities and psychosocial variables among postpartum women in Northwest Ethiopia, 2021

Categories of maternal morbidities	Number	Precent
Any maternal morbidities	282	36.4
Any direct maternal morbidities	252	32.5
Any indirect maternal morbidities	210	27.1
**Leading direct maternal morbidities**		
Complications of delivery management ^a^	140	18.1
Hypertensive disorders ^b^	57	7.4
Obstetric hemorrhage ^c^	6	0.8
Pregnancy related infections ^d^	156	20.1
**Leading indirect maternal morbidities**		
Respiratory and infectious diseases ^e^	65	8.4
Preexisting diseases before pregnancy ^f^	179	23.1
Other maternal diseases ^g^	169	21.8
**Type of mental health disorder**		
Depression	120	15.5
Anxiety	143	18.5
PTSD	75	9.7
**Social Support**		
Poor social support	227	29.3
Strong social support	294	37.9

^a^ Complications of spontaneous vaginal delivery: perineal tear, complications of episiotomy: episiotomy wound infection, complications of instrumental delivery: vaginal wall/perineal laceration, complications of caesarean section: caesarean section wound infection. ^b^ chronic hypertension, gestational hypertension, pre-eclampsia, eclampsia. ^c^ placenta previa, placental abruption, postpartum hemorrhage. ^d^ mastitis/breast abscess, chorioamnionitis, puerperal sepsis, urinary tract infection ^e^ Tuberculosis, asthma, influenza, pneumonia, malaria, HIV/AIDS, candidiasis, hepatitis. ^f^ Hypertension, anemia, tuberculosis, diabetes mellitus, hepatitis, HIV/AIDS. ^g^ Iron deficiency anemia, anemia due to vitamin B12 and/or folate deficiency

### Total functional status and domain specific scores as measured by WHODAS 2.0

The total dysfunction score was higher at the first follow up period (mean of 53.18 and SD of 22.12) and gets improved at the second (mean of 45.37 and SD of 18.03) and third (mean of 42.73 and SD of 16.36) follow up periods. With regard to domain scores, all the domains showed improvement in dysfunction score throughout the study period. The dysfunction score was higher in the community participation (mean of 11.48 and SD of 4.91) and gets slight improvement throughout the follow up period. The household life activities and mobility dysfunction scores showed better improvement than the other domains during the follow up period (see Table 3).

**Table 3:** Distribution of functional status domains at each follow up period among postpartum women in Northwest Ethiopia, 2021

Functional status domains (WHODAS 2.0)	Follow up periods		
	**6**^**th**^** week**	**12**^**th**^** week**	**18**^**th**^** week**
	Mean (SD)	Mean (SD)	Mean (SD)
Total WHODAS	53.18 (22.12)	45.37 (18.03)	42.73 (16.36)
Cognition	9.20 (4.06)	8.48 (3.53)	8.15 (3.25)
Mobility	9.34 (4.52)	7.10 (3.32)	6.48 (2.95)
Self-care	6.73 (3.21)	5.53 (2.57)	5.14 (2.13)
Getting along with people	8.09 (3.91)	7.66 (3.52)	7.33 (3.35)
Household life activities	8.26 (3.98)	6.15 (3.08)	5.54 (2.70)
Community participation	11.48 (4.91)	10.39 (3.90)	10.03 (3.70)

### Overall and domain scores of WHODAS 2.0 by maternal morbidity status

Women with direct maternal morbidities (*p* < 0.01) and women with indirect maternal morbidities (*p* < 0.01) had total dysfunction scores that were higher than women without such conditions at the three follow up periods. In addition, dysfunction scores of the domain were significantly higher in women with direct and indirect maternal morbidities. However, dysfunction score for the mobility domain, did not alter significantly between the second and third follow-up periods. Similarly, the difference between women with and without direct maternal morbidities disappears for the dysfunction score of life activity, self-care and participation domains at the 18^th^ postpartum week (see Table 4).

**Table 4:** Mean and standard deviations for WHODAS 2.0 domain and total scores by direct and indirect maternal morbidity status among postpartum women in Northwest Ethiopia, 2021

**WHODAS 2.0 domains**	**6**^**th**^ **week postpartum**				**12**^**th**^ **week postpartum**			
	**Any direct morbidity** ^**a**^		**Any indirect morbidity** ^**b**^		**Any direct morbidity** ^**a**^		**Any indirect morbidity** ^**b**^	
	**Yes**	**No**	**Yes**	**No**	**Yes**	**No**	**Yes**	**No**
**Total WHODAS: Mean (SD)**	23.73 (12.22)	12.13(15.07)	25.48(11.20)	12.37(14.96)	13.43(9.99)	8.44(13.44)	14.22(9.02)	8.53(13.42)
95%CI	22.22, 25.24	10.84, 13.43	23.96, 27.00	11.13, 13.60	12.19, 14.66	7.29, 9.60	12.99, 15.44	7.42, 9.64
*P*-value	< 0.001		< 0.001		< 0.001		< 0.001	
Cognition: Mean (SD)	10.44(3.24)	8.60(4.27)	10.83(3.18)	8.59(4.18)	8.90(2.46)	8.28(3.94)	9.09(2.46)	8.26(3.84)
95%CI	10.04, 10.84	8.23, 8.97	10.40, 11.26	8.25, 8.94	8.59, 9.20	7.95, 8.62	8.75, 9.42	7.94, 8.58
*P*-value	< 0.001		< 0.001		0.023		0.004	
Mobility: Mean (SD)	11.52(4.02)	8.30(4.38)	11.96(3.98)	8.37(4.33)	7.25(2.81)	7.03(3.54)	7.29(2.69)	9.33(4.53)
95%CI	11.02, 12.02	7.92, 8.67	11.42, 12.50	8.02, 8.73	6.90, 7.59	6.72, 7.33	6.93, 7.66	9.00, 9.65
*P*-value	< 0.001		< 0.001		0.389		0.325	
Selfcare: Mean (SD)	7.71(2.54)	6.26(3.39)	7.89(2.48)	6.30(3.34)	5.80(2.04)	5.40(2.77)	5.83(1.85)	5.42(2.78)
95%CI	7.39, 8.02	5.97, 6.55	7.55, 8.22	6.03, 6.58	5.55, 6.05	5.17, 5.64	5.58, 6.08	5.19, 5.65
*P*-value	< 0.001		< 0.001		0.045		0.046	
Getting along with people: Mean (SD)	9.91(3.55)	7.21(3.77)	10.49(3.36)	7.19(3.73)	9.33(3.26)	6.86(3.36)	9.78(3.12)	6.87(3.33)
95%CI	9.47, 10.35	6.88, 7.53	10.04, 10.95	6.89, 7.50	8.93, 9.73	6.57, 7.14	9.35, 10.20	6.60, 7.15
*P*-value	< 0.001		< 0.001		< 0.001		< 0.001	
Life activities: Mean (SD)	10.44(3.73)	7.20(3.66)	10.72(3.77)	7.34(3.66)	6.92(2.92)	5.79(3.10)	7.05(2.92)	5.82(3.08)
95%CI	9.98, 10.91	6.89, 7.51	10.21, 11.23	7.04, 7.64	6.56, 7.28	5.52(6.05)	6.65, 7.44	5.57, 6.08
*P*-value	< 0.001		< 0.001		< 0.001		< 0.001	
Participation: Mean (SD)	13.12(4.03)	10.69(5.10)	13.55(3.90)	10.72(5.03)	11.04(3.47)	10.07(4.05)	11.18(3.23)	10.09(4.08)
95%CI	12.62, 13.62	10.26, 11.13	13.02, 14.08	10.30, 11.13	10.61, 11.47	9.72, 10.42	10.74, 11.61	9.76, 10.43
*P*-value	< 0.001		< 0.001		0.001		0.001	
**18**^**th**^ **week postpartum**								
**WHODAS 2.0 domains**	**Any direct morbidity** ^**a**^				**Any indirect morbidity** ^**b**^			
	**Yes**		**No**		**Yes**		**No**	
Total WHODAS: Mean (SD)	10.03(9.83)		7.46(12.82)		10.54(8.97)		7.47(12.83)	
95%CI	8.82, 11.25		6.36, 8.56		9.32, 11.76		6.41, 8.53	
*P*-value	0.005				0.001			
Cognition: Mean (SD)	8.50(2.38)		7.98(3.58)		8.66(2.33)		7.96(3.51)	
95%CI	8.20, 8.79		7.67, 8.28		8.34, 8.97		7.67, 8.25	
*P*-value	0.037				0.007			
Mobility: Mean (SD)	6.24(2.38)		6.60(3.19)		6.17(2.14)		6.60(3.20)	
95%CI	5.95, 6.54		6.33, 6.87		5.88, 6.46		6.34, 6.86	
*P*-value	0.114				0.072			
Self-care: Mean (SD)	5.31(1.87)		5.06(2.24)		5.30(1.76)		5.08(2.25)	
95%CI	5.08, 5.54		4.86, 5.25		5.07, 5.54		4.89, 5.26	
*P*-value	0.119				0.184			
Getting along with people: Mean (SD)	8.66(3.25)		6.69(3.20)		9.04(3.19)		6.70(3.18)	
95%CI	8.26, 9.06		6.42, 6.97		8.61, 9.47		6.43, 9.96	
*P*-value	< 0.001				< 0.001			
Life activities: Mean (SD)	5.75(2.51)		5.45(2.79)		5.83(2.42)		5.44(2.80)	
95%CI	5.44, 6.06		5.21, 5.69		5.50, 6.16		5.21, 5.67	
*P*-value	0.150				0.075			
Participation: Mean (SD)	10.39(3.54)		9.86(3.76)		10.47(3.32)		9.87(3.82)	
95%CI	9.95, 10.83		9.54, 10.19		10.02, 10.92		9.56, 10.19	
*P*-value	0.063				0.047			

^**a**^ Gestational hypertension, Pre-eclampsia, Eclampsia, Placenta Previa, Placental abruption, Postpartum hemorrhage, Mastitis, Puerperal sepsis, Urinary tract infection, Perineal tear, Episiotomy wound infection, Vaginal wall/perineal laceration, Caesarean section wound infection

^**b**^ Asthma, Tuberculosis, Influenza, Pneumonia, Malaria, HIV/AIDS, Candidiasis, Hepatitis, Hypertension, Anemia, Diabetes mellitus

### Confirmatory factor analysis for WHODAS-32 items at each follow up period

In order to examine the measurement model for WHODAS -32 items at each follow up period, a confirmatory factor analysis was carried out using Stata 16.0. The various fit indices presented in Table 5 showed that the measurement model for WHODAS-32 items had a satisfactory fit for the data. The Cronbach’s alpha (α) for the internal consistency of the WHODAS-32 items also confirmed good reliability of the scale in this study (see Table 5).

**Table 5:** Results of a confirmatory factor analysis for WHODAS-32 item showing model fitness criteria and reliability test results with P-value, among postpartum women in Northwest Ethiopia, 2021

Domains	Model fitness criteria				Reliability test
	CFI	TLI	RMSEA	SRMR	Cronbach’s alpha (α)
Cognition at T1	0.762	0.737	0.096	0.104	0.899
Mobility at T1					0.933
Selfcare at T1					0.850
Getting along with people at T1					0.837
Life activities at T1					0.955
Participation at T1					0.899
Cognition at T2	0.750	0.724	0.087	0.138	0.885
Mobility at T2					0.926
Selfcare at T2					0.830
Getting along with people at T2					0.838
Life activities at T2					0.937
Participation at T2					0.903
Cognition at T3	0.735	0.707	0.082	0.139	0.875
Mobility at T3					0.929
Selfcare at T3					0.798
Getting along with people at T3					0.847
Life activities at T3					0.925
Participation at T3					0.912

**Fig. 1:**
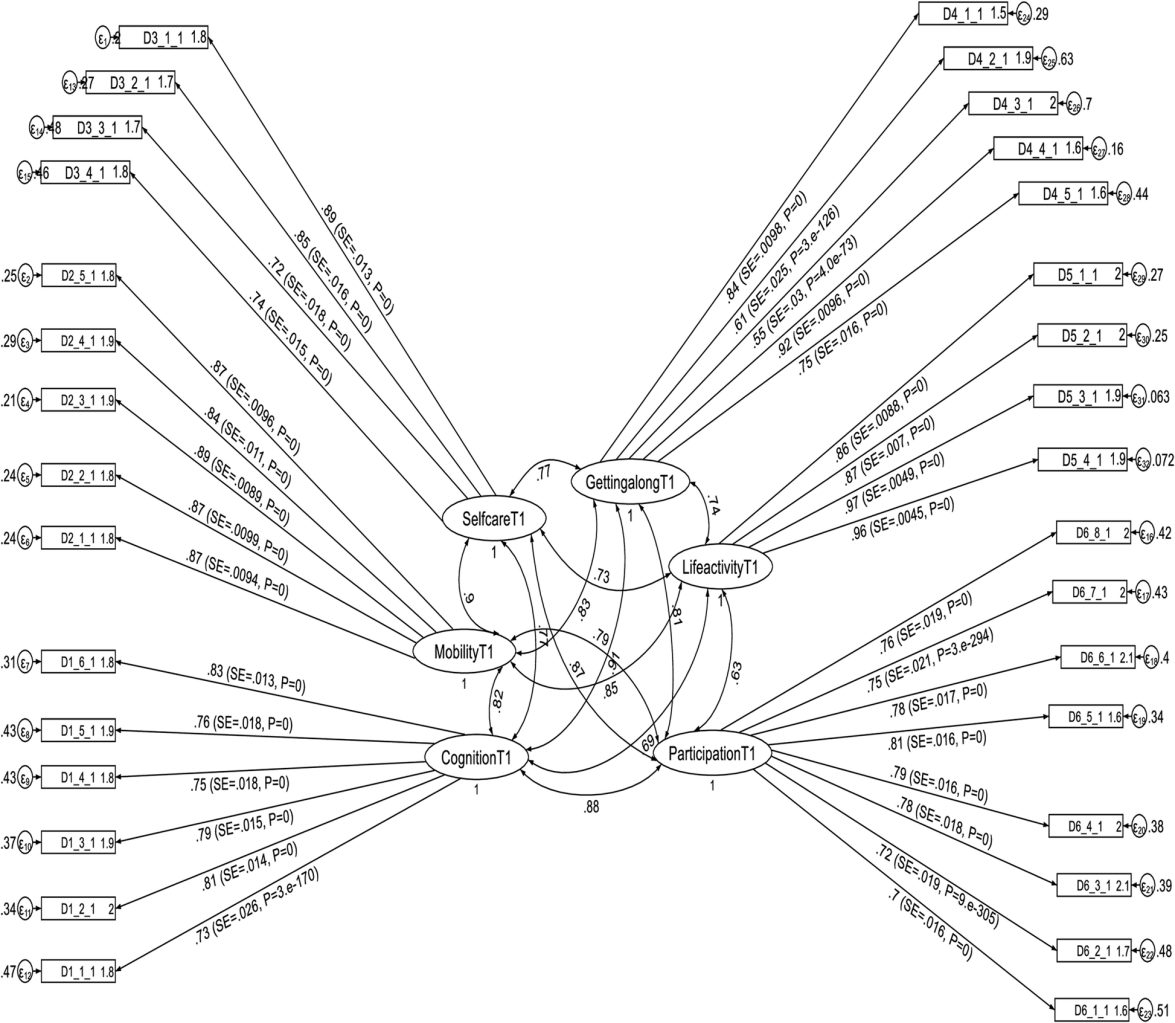
Results of a standardized factor loadings of a measurement model for the WHODAS 2.0 at the first follow up period in postpartum women, Northwest Ethiopia, 2021. Note: β’s are standardized coefficients with their SE and *P*-values

**Fig. 2:**
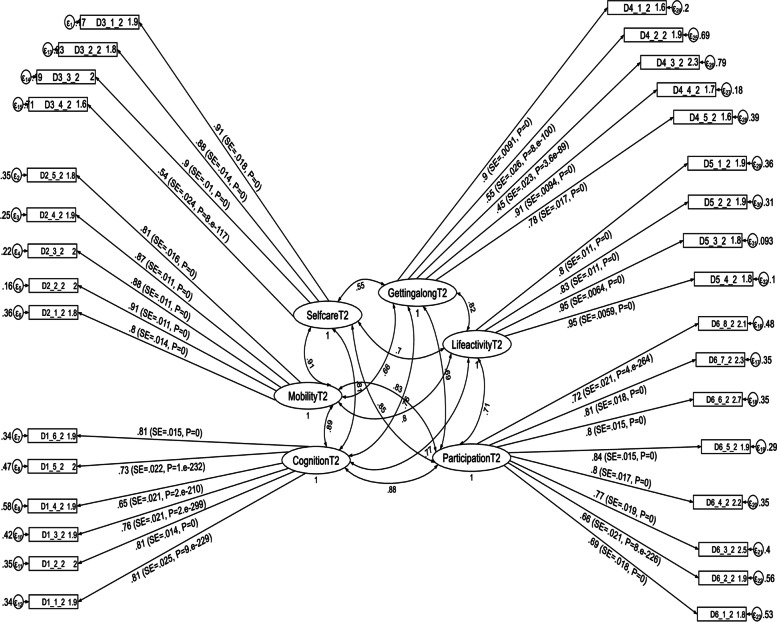
Results of a standardized factor loadings of a measurement model for the WHODAS 2.0 at the second follow up period in postpartum women, Northwest Ethiopia, 2021. Note: β’s are standardized coefficients with their SE and *P*-values

**Fig. 3:**
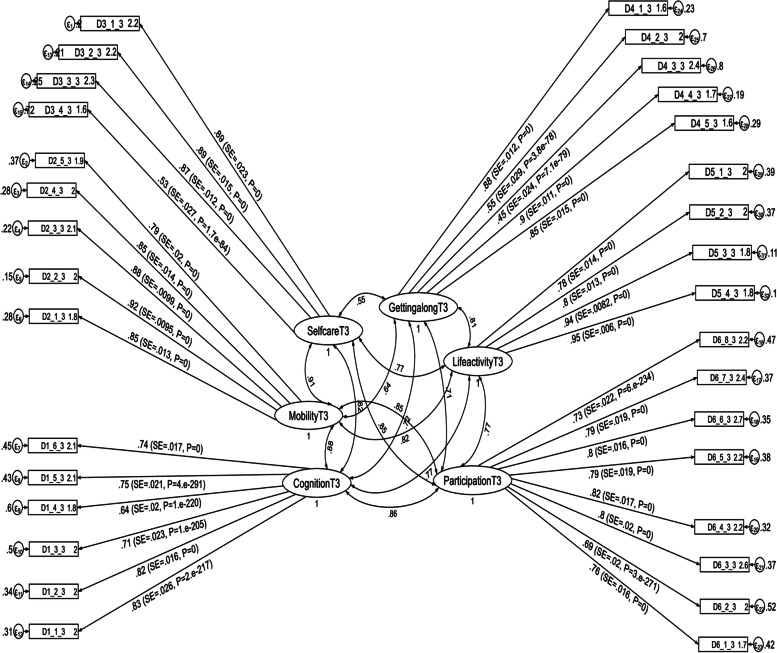
Results of a standardized factor loadings of a measurement model for the WHODAS 2.0 at the third follow up period in postpartum women, Northwest Ethiopia, 2021. Note: β’s are standardized coefficients with their SE and *P*-values

Factor loading values for each of the six domain scores ranged from 0.55 (for getting along with people domain) to 0.97 (for household life activity domain) at the first follow up period, and were significant at *p* < 0.001. The estimated standardized path loadings for the structural equation model at the first follow up period are shown in Fig. 1. At the second follow up period, factor loading values for each of the four domain scores ranged from 0.45 (for getting along with people domain) to 0.95 (for household life activity domain), and were significant at *p* < 0.001. The estimated standardized path loadings for the structural equation model at the second follow up period are shown in Fig. 2. Similarly, factor loading values for each of the six domain scores ranged from 0.45 (for getting along with people domain) to 0.95 (for household life activity domain) at the third follow up period, and were significant at *p* < 0.001. The estimated standardized path loadings for the structural equation model at the third follow up period are shown in Fig. 3.

### Direct, indirect and total effects of variables associated with functional status at each follow up period

In order to examine the direct and indirect association of variables with functional status at each follow up period, we have carried out a longitudinal path analyses using a linear structural equation model. The model fits the data well according to various fit indices. The model fitness indices for the cognition domain were: CFI = 0.995, TLI = 0.963, RMSEA = 0.088 and SRMR = 0.018. The model fitness indices for the mobility domain were: CFI = 0.989, TLI = 0.949, RMSEA = 0.084 and SRMR = 0.058. The model fitness indices for the selfcare domain were: CFI = 0.990, TLI = 0.952, RMSEA = 0.084 and SRMR = 0.059. The model fitness indices for the getting along with people domain were: CFI = 0.992, TLI = 0.963, RMSEA = 0.084 and SRMR = 0.059. The model fitness indices for the household life activity domain were: CFI = 0.995, TLI = 0.967, RMSEA = 0.067 and SRMR = 0.014. The model fitness indices for the community participation domain were: CFI = 0.997, TLI = 0.977, RMSEA = 0.067 and SRMR = 0.015. The model fitness indices for the total WHODAS score were: CFI = 0.992, TLI = 0.961, RMSEA = 0.084 and SRMR = 0.062.

**Fig. 4:**
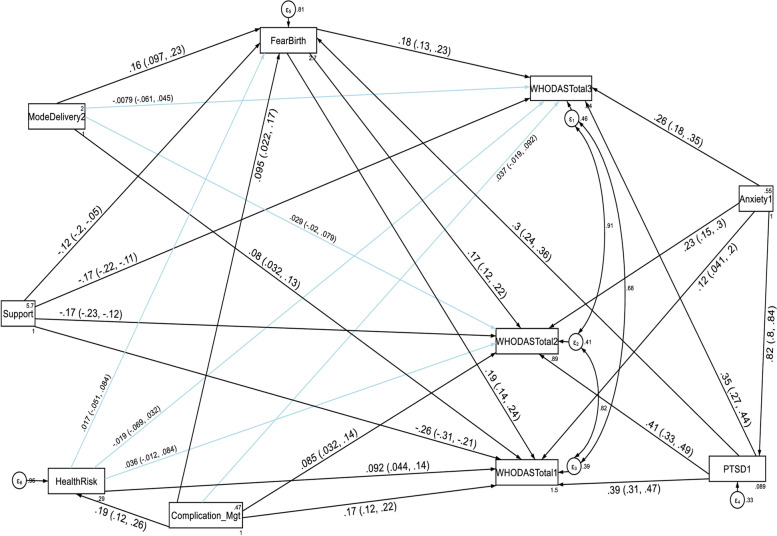
A structural equation model of the factors associated with the WHODAS total score in postpartum women, Northwest Ethiopia. Note: β’s are standardized estimates with 95% CI

Higher fear of childbirth score, anxiety and PTSD score had a direct deleterious effect (increased risk of functional disability) on the total functional disability. Higher PTSD symptom score had also an indirect deleterious effect (increased risk of functional disability) through fear of birth (by increasing fear of birth score) on the total functional disability score throughout the follow up period (See Table 6 and Fig. 4). Complications of delivery management increased the WHODAS total disability score of women by 6.67 and 2.77 at the first and second follow up periods respectively, compared to women without delivery management complications (See Figs. 4 and Table 6). Contrary to this, higher social support had a direct protective effect (decreased risk of functional disability) on the total functional disability score at the three follow up periods. It had also an indirect protective effect through fear of birth (by decreasing fear of birth score) on the total functional disability score throughout the follow up period (see Table 6 and Fig. 4).

**Table 6:** Direct, indirect and total effects of variables associated with WHODAS total score and the cognition domain among postpartum women, Northwest Ethiopia, 2021

**Variable’s pathway**	**WHODAS 2.0 domains at each follow up period**											
	**T1 WHODAS total score**			**T2 WHODAS total score**				**T3 WHODAS total score**				
	Direct effect	Indirect effect	Total effect	Direct effect	Indirect effect	Total effect		Direct effect		Indirect effect		Total effect
	β (SE)	β (SE)	β (SE)	β (SE)	β (SE)	β (SE)		β (SE)		β (SE)		β (SE)
**Fear of birth**	0.11(0.01) ^a^	No path	0.11(0.01) ^a^	0.08(0.01) ^a^	No path	0.08(0.01) ^a^		0.08(0.01) ^a^		No path		0.08(0.01) ^a^
**Mode of delivery SVD**	1.78 (0.55) ^b^	0.69(0.17) ^a^	2.46(0.56) ^a^	Not significant	0.52(0.14) ^a^	1.06(0.48) ^b^		Not significant		0.53(0.14) ^a^		Not significant
**Anxiety score**	0.30(0.09) ^a^	0.84(0.08) ^a^	1.11(0.06) ^a^	0.43(0.08) ^a^	0.73(0.07) ^a^	1.16(0.05) ^a^		0.50(0.08) ^a^		0.61(0.07) ^a^		1.10(0.05) ^a^
**PTSD score**	0.46(0.05) ^a^	No path	0.54(0.05) ^a^	0.41(0.04) ^a^	No path	0.47(0.04) ^a^		0.34(0.04) ^a^		No path		0.40(0.04) ^a^
**Social support**	-2.14(0.20) ^a^	-0.33(0.06) ^a^	-2.47(0.20) ^a^	-1.09(0.17) ^a^	-0.23(0.05) ^a^	-1.32(0.17) ^a^		-0.90(0.17) ^a^		-0.23(0.05) ^a^		-1.13(0.17) ^a^
**Delivery Mgt complicationYes**	6.67(1.02) ^a^	1.412(0.36) ^a^	8.08(1.06) ^a^	2.77(0.88) ^b^	0.77(0.27) ^b^	3.54(0.89) ^a^		Not significant		Not significant		Not significant
**Health risk Yes**	4.19(1.09) ^a^	Not significant	4.33(1.12) ^a^	Not significant	Not significant	3.09(0.84) ^a^		Not significant		Not significant		Not significant
**Variable’s pathway**	**WHODAS 2.0 domains at each follow up period**											
	**T1 cognition domain**			**T2 cognition domain**			**T3 cognition domain**					
	Direct effect	Indirect effect	Total effect	Direct effect	Indirect effect	Total effect	Direct effect		Indirect effect		Total effect	
	β (SE)	β (SE)	β (SE)	β (SE)	β (SE)	β (SE)	β (SE)		β (SE)		β (SE)	
**Fear of birth**	0.02(0.004) ^a^	No path	0.02(0.004) ^a^	0.01(0.004) ^a^	No path	0.01(0.004) ^a^	0.01(0.003) ^b^		No path		0.01(0.003) ^b^	
**Mental quality of life score**	-0.04(0.01) ^a^	-0.01(0.002) ^b^	-0.04(0.01) ^a^	-0.03(0.01) ^a^	Not significant	-0.03(0.01) ^a^	-0.03(0.01) ^a^		Not significant		-0.03(0.01) ^a^	
**Anxiety score**	0.10(0.03) ^a^	0.26(0.02) ^a^	0.35(0.02) ^b^	0.13(0.02) ^a^	0.2(0.02) ^a^	0.35(0.01) ^a^	0.16(0.02) ^a^		0.16(0.02) ^a^		0.31(0.01) ^a^	
**PTSD score**	0.15(0.01) ^a^	No path	0.17(0.02) ^a^	0.13(0.01) ^a^	No path	0.14(0.01) ^a^	0.09(0.01) ^a^		No path		0.10(0.01) ^a^	
**Social support**	-0.54(0.05) ^a^	-0.07(0.02) ^a^	-0.61(0.05) ^a^	-0.26(0.05) ^a^	-0.03(0.01) ^b^	-0.29(0.04) ^a^	-0.24(0.04) ^a^		-0.03(0.01) ^b^		-0.27(0.04) ^a^	
**Primary & above** **education** Yes	-0.24(0.08) ^b^	No path	-0.23(0.08) ^b^	-0.20(0.07) ^b^	No path	-0.19(0.07) ^b^	-0.15(0.07) ^b^		No path		-0.15(0.07) ^b^	
**Delivery Mgt** **complication** **Yes**	1.52(0.28) ^a^	No path	1.72(0.27) ^a^	0.81(0.25) ^a^	No path	0.90(0.25) ^a^	0.66(0.25) ^b^		No path		0.74(0.25) ^b^	

^a^ *p*-value ≤ 0.001, ^b^ *p*-value < 0.05, β is unstandardized estimate, SVD is spontaneous vaginal delivery, Delivery management complication includes; perineal tear, episiotomy wound infection, Cesarean section wound infection

**Fig. 5:**
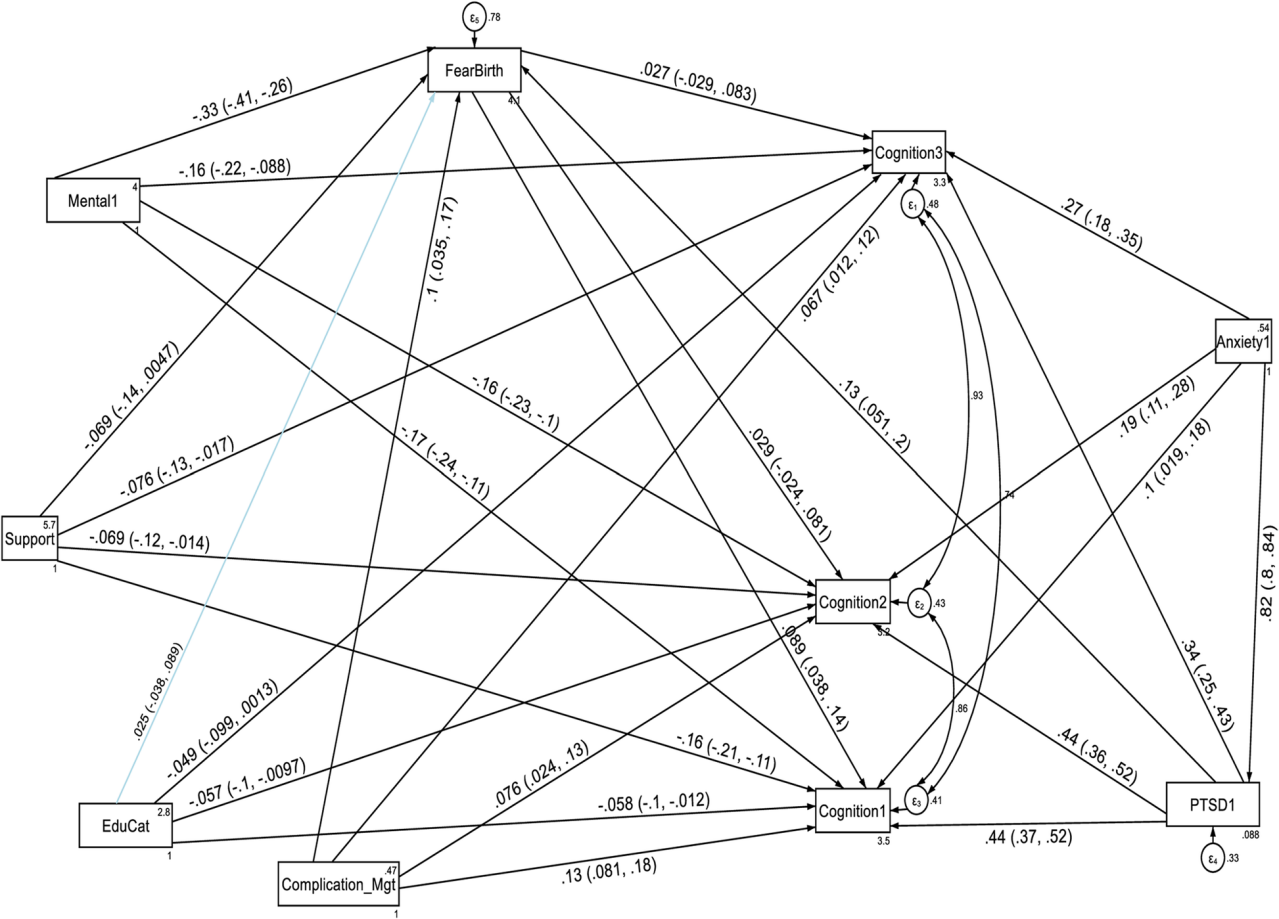
A structural equation model of the factors associated with cognition domain of WHODAS 2.0 in postpartum women, Northwest Ethiopia. Note: β’s are standardized estimates with 95% CI

**Fig. 6:**
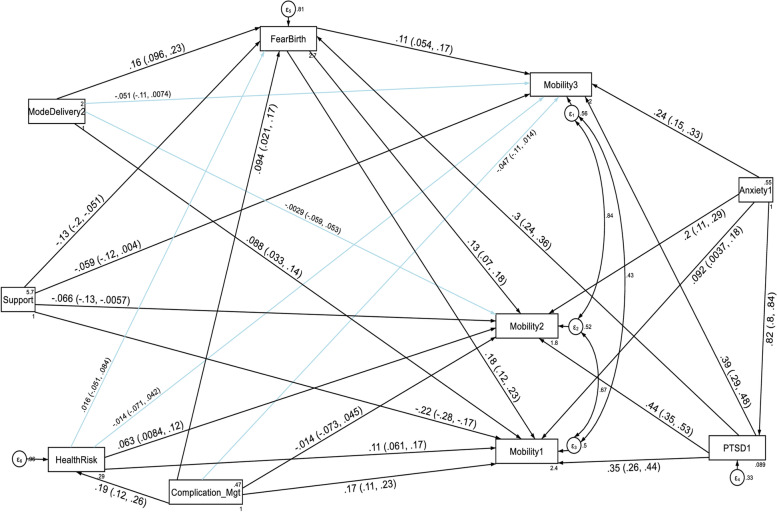
A structural equation model of the factors associated with mobility domain of WHODAS 2.0 in postpartum women, Northwest Ethiopia. Note: β’s are standardized estimates with 95% CI

**Fig. 7:**
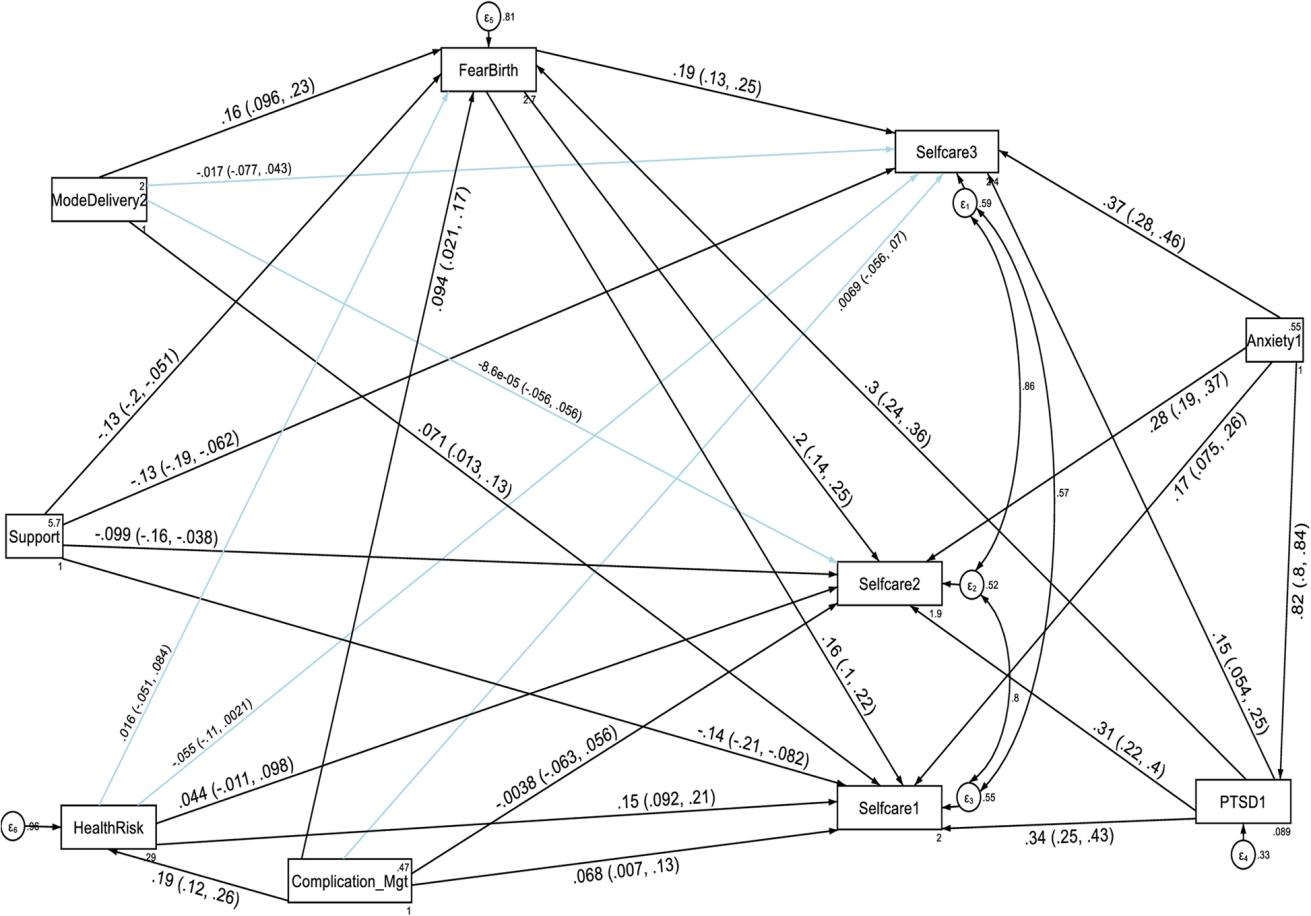
A structural equation model of the factors associated with selfcare domain of WHODAS 2.0 in postpartum women, Northwest Ethiopia. Note: β’s are standardized estimates with 95% CI

**Fig. 8:**
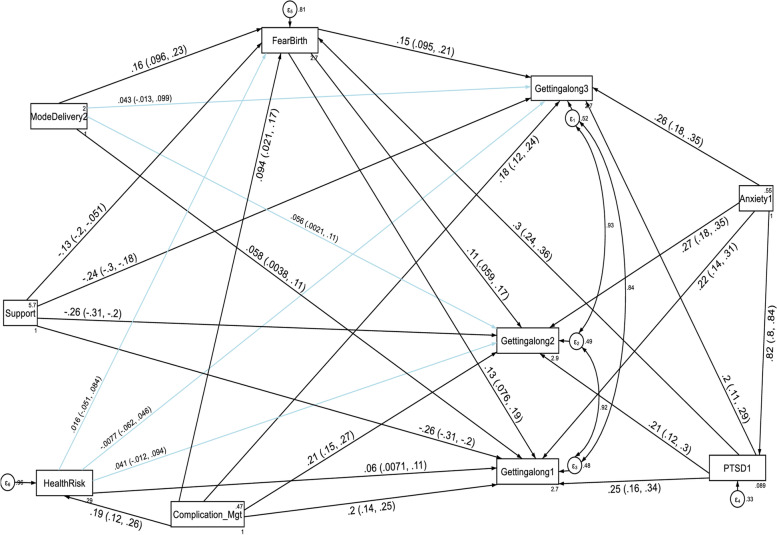
A structural equation model of the factors associated with the getting along with people domain of WHODAS 2.0 in postpartum women, Northwest Ethiopia. Note: β’s are standardized estimates with 95% CI

**Fig. 9:**
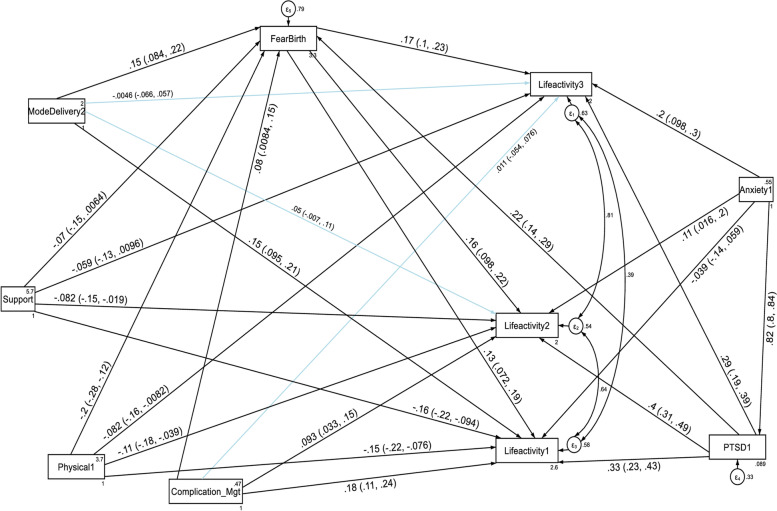
A structural equation model of the factors associated with the household life activities domain of WHODAS 2.0 in postpartum women, Northwest Ethiopia. Note: β’s are standardized estimates with 95% CI

**Fig. 10:**
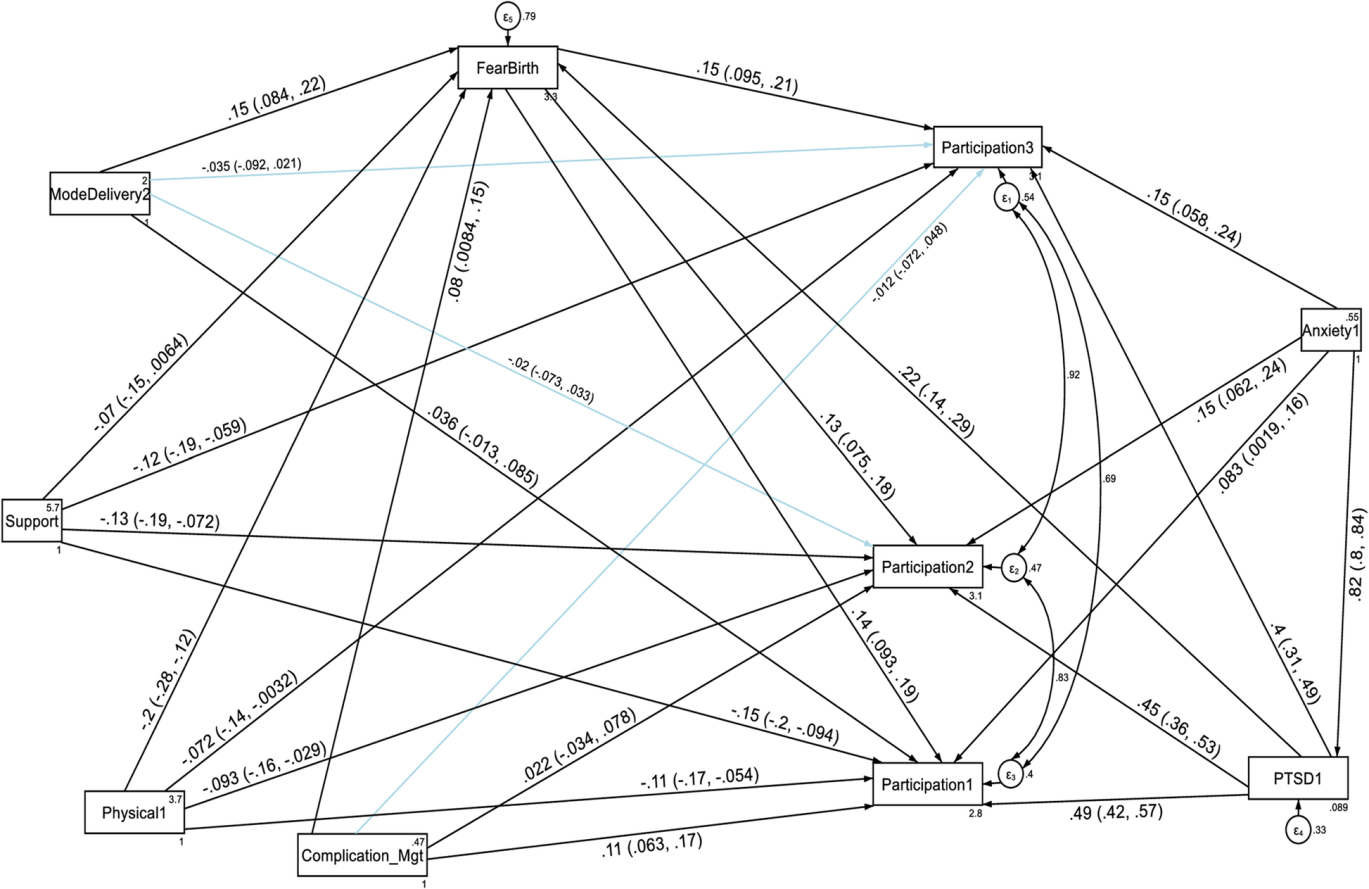
A structural equation model of the factors associated with the community participation domain of WHODAS 2.0 in postpartum women, Northwest Ethiopia. Note: β’s are standardized estimates with 95% CI

With regard to the domain scores of functional disabilities, higher fear of childbirth score, anxiety and PTSD had a direct deleterious effect (increased risk of functional disability) on the six domains of WHODAS 2.0 (cognition, mobility, self-care, getting along with people, household life activities and community participation) at the first, second and third follow up periods. Higher PTSD symptom score had also an indirect deleterious effect (increased risk of functional disability) through fear of birth (by increasing fear of birth score) on the six domains of functional disability score throughout the follow up period (See Fig. 5, 6, 7, 8, 9, and 10, Tables 6, 7 and 8). Whereas, higher social support had a direct protective effect (decreased risk of functional disability) on the functional disability score of all the domains at the three follow up periods. It had also an indirect protective effect through fear of birth (by decreasing fear of birth score) on these domains of WHODAS 2.0 throughout the follow up period. Higher mental quality of life score was also found to be protective of functional disability for the cognition domain (see Figs. 5, 6, 7, 8, 9, and 10, Tables 6, 7 and 8).

**Table 7:** Direct, indirect and total effects of variables associated with the mobility, selfcare and getting along with people domains of WHODAS 2.0 among postpartum women, Northwest Ethiopia, 2021

**Variable’s pathway**	**WHODAS 2.0 domains at each follow up period**																	
	**T1 mobility domain**						**T2 mobility domain**						**T3 mobility domain**					
	Direct effect		Indirect effect		Total effect		Direct effect		Indirect effect		Total effect		Direct effect		Indirect effect		Total effect	
	β (SE)		β (SE)		β (SE)		β (SE)		β (SE)		β (SE)		β (SE)		β (SE)		β (SE)	
**Fear of birth**	0.03(0.005) ^a^		No path		0.03(0.005) ^a^		0.02(0.004) ^a^		No path		0.02(0.004) ^a^		0.01(0.003) ^a^		No path		0.01(0.003) ^a^	
**Mode of delivery** **SVD**	0.57(0.18) ^a^		0.19(0.05) ^a^		0.76(0.18) ^a^		Not significant		0.10(0.03) ^b^		Not significant		Not significant		0.08(0.03) ^b^		Not significant	
**Anxiety score**	0.06(0.03) ^b^		0.22(0.03) ^a^		0.29(0.02) ^a^		0.10(0.02) ^a^		0.20(0.02) ^a^		0.30(0.02) ^a^		0.11(0.02) ^a^		0.16(0.02) ^a^		0.27(0.01) ^a^	
**PTSD score**	0.12(0.02) ^a^		No path		0.14(0.02) ^a^		0.12(0.01) ^a^		No path		0.13(0.01) ^a^		0.09(0.01) ^a^		No path		0.10(0.01) ^a^	
**Social support**	-0.58(0.07) ^a^		-0.09(0.02) ^a^		-0.67(0.07) ^a^		-0.12(0.05) ^b^		-0.04(0.01) ^a^		-0.16(0.05) ^a^		-0.07(0.05) ^c^		-0.03(0.01) ^b^		-0.10(0.05) ^b^	
**Delivery Mgt complication** **Yes**	1.95(0.34) ^a^		No path		2.40(0.35) ^a^		Not significant		No path		Not significant		Not significant		No path		Not significant	
**Health risk: Yes**	2.31(0.33) ^a^		No path		2.31(0.33) ^a^		Not significant		No path		Not significant		Not significant		No path		Not significant	
**Variable’s pathway**		**WHODAS 2.0 domains at each follow up period**																
		**T1 selfcare domain**						**T2 selfcare domain**						**T3 selfcare domain**				
		Direct effect		Indirect effect		Total effect		Direct effect		Indirect effect		Total effect		Direct effect		Indirect effect		Total effect
		β (SE)		β (SE)		β (SE)		β (SE)		β (SE)		β (SE)		β (SE)		β (SE)		β (SE)
**Fear of birth**		0.02(0.004) ^a^		No path		0.02(0.004) ^a^		0.02(0.003) ^a^		No path		0.02(0.003) ^a^		0.02(0.003) ^a^		No path		0.02(0.003) ^a^
**Mode of delivery SVD**		0.33(0.14) ^b^		0.12(0.04) ^a^		0.45(0.14) ^a^		Not significant		Not significant		Not significant		Not significant		Not significant		Not significant
**Anxiety score**		0.08(0.02) ^a^		0.15(0.02) ^a^		0.23(0.01) ^a^		0.01(0.02) ^a^		0.12(0.02) ^a^		0.23(0.01) ^a^		0.12(0.02) ^a^		0.06(0.01) ^a^		0.20(0.01) ^a^
**PTSD score**		0.09(0.01) ^a^		0.01(0.004) ^a^		0.10(0.01) ^a^		0.06(0.01) ^a^		0.01(0.003) ^a^		0.08(0.01) ^a^		0.03(0.01) ^b^		0.01(0.003) ^a^		0.04(0.01) ^a^
**Social support**		-0.29(0.05) ^a^		-0.06(0.01) ^a^		-0.35(0.05) ^a^		-0.13(0.04) ^a^		-0.05(0.01) ^a^		-0.18(0.04) ^a^		-0.12(0.03) ^a^		-0.04(0.01) ^a^		-0.16(0.04) ^a^
**Delivery Mgt complication Yes**		0.56(0.26) ^b^		No path		0.92(0.26) ^a^		Not significant		No path		Not significant		Not significant		No path		Not significant
**Health risk Yes**		1.42(0.27) ^a^		No path		1.44(0.28) ^a^		Not significant		No path		Not significant		Not significant		No path		Not significant
**Variable’s pathway**		**WHODAS 2.0 domains at each follow up period**																
		**T1 getting along with people domain**						**T2 getting along with people domain**						**T3 getting along with people domain**				
		Direct effect		Indirect effect		Total effect		Direct effect		Indirect effect		Total effect		Direct effect		Indirect effect		Total effect
		β (SE)		β (SE)		β (SE)		β (SE)		β (SE)		β (SE)		β (SE)		β (SE)		β (SE)
**Fear of birth**		0.02(0.004) ^a^		No path		0.02(0.004) ^a^		0.17(0.004) ^a^		No path		0.17(0.004) ^a^		0.02(0.004) ^a^		No path		0.02(0.004) ^a^
**Mode of delivery SVD**		0.33(0.16) ^b^		0.12(0.04) ^a^		0.45(0.16) ^b^		0.29(0.14) ^b^		0.10(0.03) ^b^		0.39(0.14) ^b^		Not significant		0.12(0.04) ^a^		0.33(0.14) ^b^
**Anxiety score**		0.13(0.03) ^a^		0.14(0.02) ^a^		0.28(0.02) ^a^		0.14(0.02) ^a^		0.11(0.02) ^a^		0.25(0.01) ^a^		0.14(0.02) ^a^		0.10(0.02) ^a^		0.24(0.01) ^a^
**PTSD score**		0.08(0.01) ^a^		No path		0.09(0.01) ^a^		0.06(0.01) ^a^		No path		0.07(0.01) ^a^		0.05(0.01) ^a^		No path		0.06(0.01) ^a^
**Social support**		-0.51(0.06) ^a^		-0.06(0.02) ^a^		-0.56(0.06) ^a^		-0.45(0.05) ^a^		-0.05(0.01) ^a^		-0.50(0.05) ^a^		-0.39(0.05) ^a^		-0.06(0.01) ^a^		-0.45(0.05) ^a^
**Delivery Mgt complication Yes**		2.0(0.30) ^a^		No path		2.23(0.30) ^a^		1.91(0.27) ^a^		No path		2.08(0.27) ^a^		1.55(0.27) ^a^		No path		1.67(0.27) ^a^
**Health risk Yes**		0.70(0.31) ^b^		No path		0.73(0.32) ^b^		Not significant		No path		Not significant		Not significant		No path		Not significant

^a^ *p*-value ≤ 0.001, ^b^ *p*-value < 0.05, β is unstandardized estimate, SVD is spontaneous vaginal delivery, Delivery management complication includes; perineal tear, episiotomy wound infection, Cesarean section wound infection

**Table 8:** Direct, indirect and total effects of variables associated with the household life activities and community participation domains of WHODAS 2.0 among postpartum women, Northwest Ethiopia, 2021

**Variable’s pathway**	**WHODAS 2.0 domains at each follow up period**								
	**T1 household life activities domain**			**T2 household life activities domain**			**T3 household life activities domain**		
	Direct effect	Indirect effect	Total effect	Direct effect	Indirect effect	Total effect	Direct effect	Indirect effect	Total effect
	β (SE)	β (SE)	β (SE)	β (SE)	β (SE)	β (SE)	β (SE)	β (SE)	β (SE)
**Fear of birth**	0.03(0.005) ^a^	No path	0.03(0.005) ^a^	0.02(0.003) ^a^	No path	0.02(0.003) ^a^	0.02(0.003) ^a^	No path	0.02(0.003) ^a^
**Mode of delivery SVD**	0.93(0.18) ^a^	No path	0.93(0.18) ^a^	Not significant	No path	Not significant	Not significant	No path	Not significant
**Anxiety score**	Not significant	0.20(0.03) ^a^	0.20(0.02) ^a^	0.07(0.02) ^b^	0.18(0.02) ^a^	0.25(0.01) ^a^	0.09(0.02) ^b^	0.12(0.02) ^a^	0.21(0.01) ^b^
**PTSD score**	0.12(0.02) ^a^	No path	0.14(0.02) ^a^	0.10(0.01) ^a^	No path	0.11(0.01) ^a^	0.07(0.01) ^a^	No path	0.08(0.01) ^a^
**Social support**	-0.46(0.06) ^a^	-0.07(0.02) ^a^	-0.53(0.06) ^a^	-0.18(0.05) ^a^	-0.06(0.01) ^a^	-0.24(0.05) ^a^	-0.10(0.04) ^b^	-0.05(0.01) ^a^	-0.15(0.04) ^a^
**Physical quality of life score**	-0.03(0.01) ^a^	-0.01(0.002) ^a^	-0.04(0.01) ^a^	-0.02(0.01) ^b^	-0.01(0.001) ^a^	-0.03(0.01) ^a^	-0.01(0.005) ^b^	-0.01(0.001) ^a^	-0.02(0.01) ^b^
**Delivery Mgt complicationYes**	1.94(0.33) ^a^	No path	2.16(0.34) ^a^	0.82(0.25) ^a^	No path	1.00(0.25) ^a^	Not significant	No path	Not significant
**Variable’s pathway**	**WHODAS 2.0 domains at each follow up period**								
	**T1 community participation domain**			**T2 community participation domain**			**T3 community participation domain**		
	Direct effect	Indirect effect	Total effect	Direct effect	Indirect effect	Total effect	Direct effect	Indirect effect	Total effect
	β (SE)	β (SE)	β (SE)	β (SE)	β (SE)	β (SE)	β (SE)	β (SE)	β (SE)
**Fear of birth**	0.03(0.005) ^a^	No path	0.03(0.005) ^a^	0.02(0.004) ^a^	No path	0.02(0.004) ^a^	0.02(0.004) ^a^	No path	0.02(0.004) ^a^
**Mode of delivery** **SVD**	Not significant	0.16(0.05) ^a^	0.41(0.18) ^b^	Not significant	0.11(0.04) ^b^	Not significant	Not significant	0.13(0.04) ^a^	Not significant
**Anxiety score**	0.06(0.03) ^b^	0.32(0.03) ^a^	0.39(0.02) ^a^	0.09(0.03) ^a^	0.23(0.02) ^a^	0.32(0.02) ^a^	0.09(0.03) ^b^	0.20(0.02) ^a^	0.29(0.02) ^a^
**PTSD score**	0.21(0.02) ^a^	No path	0.23(0.02) ^a^	0.15(0.01) ^a^	No path	0.16(0.01) ^a^	0.12(0.01) ^a^	No path	0.14(0.01) ^a^
**Social support**	-0.44(0.07) ^a^	-0.09(0.02) ^a^	-0.53(0.07) ^a^	-0.28(0.06) ^a^	-0.06(0.01) ^a^	-0.34(0.06) ^a^	-0.24(0.06) ^a^	-0.06(0.02) ^a^	-0.30(0.06) ^a^
**Delivery Mgt complication Yes**	1.46(0.34) ^a^	No path	1.61(0.34) ^a^	Not significant	No path	Not significant	Not significant	No path	Not significant
**Physical quality of life score**	-0.03(0.01) ^a^	No path	-0.04(0.01) ^a^	-0.02(0.01) ^b^	No path	-0.02(0.01) ^a^	-0.01(0.006) ^b^	No path	-0.02(0.01) ^b^

^a^ *p*-value ≤ 0.001, ^b^ *p*-value < 0.05, β is unstandardized estimate, SVD is spontaneous vaginal delivery, Delivery management complication includes; perineal tear, episiotomy wound infection, Cesarean section wound infection

Complications of delivery management had a direct deleterious effect on the disability score of getting along with people domain at the three follow up periods. It increases the getting along with people disability score of women by 2.0, 2.23 and 2.08 at T1, T2 and T3 respectively compared to women without delivery management complications (See Table 7 and Fig. 8). Complications of delivery management had also a direct deleterious effect on the disability score of the household life activities domain at the first and second follow up periods. It increases the household life activities disability score of women by 1.94 and 0.82 at T1 and T2 respectively compared to women without delivery management complications (See Table 8 and Fig. 9). 

Complications of delivery management had also a direct deleterious effect on the disability score of mobility, self-care and community participation domains at the first follow up period (See Table 6, 7 and 8). It increased the community participation disability score of women by 1.46 compared to women without delivery management complications (see Table 8 and Fig. 10). Higher physical quality of life score was found to be protective of functional disability for the household life activity and community participation domains (see Table 8, Fig. 9 and 10). 

## Discussion

Findings of this study build upon results of a recent study examining predictors of functional status trajectory group membership at 6, 12 and 18 weeks postpartum [4]. The current study examined pathways of risk factors for postnatal functional impairment in women across three waves of data, from 6 to 18 weeks postpartum. The results highlight the importance of mediating role of postnatal risk factors, namely, fear of childbirth, posttraumatic stress disorder and life-threatening event of health risk, in predicting postnatal functional status impairment. The current study replicated the mediating role of fear of childbirth, posttraumatic stress disorder, and health risk for the association of maternal functioning in postpartum period with complications of delivery management, poor social support, vaginal mode of delivery, anxiety, physical quality of life and mental quality of life and expanded upon these results by evaluating these effects at several time points. 

Results indicated that fear of child birth mediated the association of all domains of functional impairment with complications of delivery management, social support, mode of delivery, anxiety, PTSD, physical quality of life and mental quality of life at 6, 12 and 18 weeks postpartum. All these factors were found to be directly associated with the poor functional status trajectory group membership in a previous publication [4], but the causal pathways (mediating relationship) of these variables were not investigated in the previous publication. Life threatening event of health risk also mediated the association of delivery management complications with all domains of functional impairment except the cognition and life activity domains at 6, 12 and 18 weeks postpartum. Similarly, the association of anxiety with all domains of functional status was also mediated by posttraumatic stress disorder symptoms during the postpartum period. These findings elucidate how these risk pathways contribute to the course of functional impairment during the first 18 weeks after childbirth. 

In this study, the total functional disability scores were found to be 53.18, 45.37 and 42.73 at the 6^th^, 12^th^ and 18^th^ week of postpartum period indicating an improvement over time. The domain scores of functional disabilities were also found to be improved over the follow up period in this study. The total and domain specific functional disability scores of this study were higher than the findings of previous studies [14, 15, 37–42]. These might be due to differences in the socio-economic status of the study population and timing of functional status measurement. 

Findings of this study indicated that fear of child birth, life threatening event of health risk and PTSD not only directly predicted poor functional status group membership [4], but also mediated the effects of delivery management complications (which were among the direct maternal morbidities), social support, mode of delivery, anxiety, physical and mental quality of life which could indicate the causal pathway of functional impairment. Therefore, interventions targeting at prevention of fear of child birth, life threatening event of health risk and PTSD could avert the possible negative effects of complications of delivery management, poor social support, vaginal mode of delivery, anxiety, poor physical and mental quality of life on functional status of postpartum women.

Though other studies had been conducted to explore the direct association of predictors with functional impairment [4, 6, 7, 10–12, 14, 16, 42], this is the first study, to the author’s knowledge, to examine the mediating role (indirect effect) of key psychosocial risk factors of functional impairment using a structural equation modelling which could indicate the possible pathways of causation, so that an intervention could be made at a certain path to halt the next unfavorable outcome (functional impairment). As such, this pattern of findings could reflect the relative importance of fear of childbirth, PTSD and health risk in explaining the path for the association of functional status with complications of delivery management, social support, mode of delivery, anxiety, physical and mental quality of life.

In a previous publication, it is found that women with anxiety symptoms are more likely to develop PTSD symptoms [20]. The current results indicated that women with PTSD symptoms because of anxiety symptoms will continue to develop functional impairment. So that, interventions targeting at reduction of anxiety symptoms could prevent the double burden of anxiety which is PTSD and functional impairment. As supported by the results of a linear structural equation modelling in this study, higher social support was also found to have an indirect protective association with all domains of functional status (decreased functional disability scores) as measured by WHODAS-32 items through fear of childbirth (by reducing fear of childbirth scores). Therefore, interventions targeting at mechanisms of improving social support could enhance the functional status of mothers in the postpartum period. 

In addition, the linear structural equation modeling results of this study also showed that, complications of delivery management (perineal tear, vaginal wall/perineal laceration, episiotomy and cesarean section wound infection), were found to have a direct deleterious association with all domains of WHODAS 2.0 (increased functional disability score). These delivery management complications were among the obstetric complications. These results are in line with earlier researches that showed women with clinically recognized medical and obstetric complications were generally more likely to function at a lower level than those without a morbidity [12, 14]. The linear structural equation modelling results of this study, also supported that anxiety and PTSD symptoms had a direct deleterious association with all domains of WHODAS 2.0 (increased functional disability scores). In addition, PTSD symptoms were found to have an indirect deleterious association with all domains of the functional status measured by WHODAS-32 items through fear of childbirth. These findings are congruent with previous literatures which reported that women with anxiety and PTSD symptoms were at a higher risk of daily functioning impairment [43–45].

### Strength and limitation of the study

To the author's knowledge, this is the first study to explore the direct and indirect causal mechanisms of the factors associated with functional status scores as measured by WHODAS 2.0 among postpartum women in Ethiopia. Through a structural equation modelling frame of analysis, the direct, indirect and total effects of predictor variables were measured with adequate sample size and a low attrition rate. This method allowed to investigate both the direct and indirect relationships between several postulated risk factors and functional impairment.

The study had also limitations. First, most variables were self-report and it is possible that women might not be comfortable in disclosing information about their own levels of stress, anxiety, depression and PTSD because of concerns about social desirability. It was unable to evaluate the subdomain of the functional status questionnaire that measures occupational life activities because employee women were on maternity leave at the time of data collection.

## Conclusion

Maternal functioning in the postpartum period is initially impaired, but improves over time. Despite improvement, maternal morbidities are correlated with worse functioning scores compared to women without these morbidities. The effect of complications of delivery management, anxiety, vaginal mode of delivery, poor social support, physical and mental quality of life on functional impairment were mediated by fear of child birth, life threatening event of health risk and PTSD symptom. Complications of delivery management, fear of childbirth, anxiety and PTSD symptoms had a direct and indirect deleterious effect on the six domains of functional status and total functional disability scores at the first, second and third follow up period. Whereas, higher social support scale had a direct and indirect protective effect on these domains of functional disability scores at the three follow up periods.

### Recommendation

One option for decreasing the functional impairment gap and enhancing the ability of the mother to resume regular social and occupational activities is the diagnosis and treatment of delivery-related complications, anxiety, and PTSD during the postpartum period. Interventions targeting at prevention of fear of child birth, life threatening event of health risk and PTSD could also avert the possible negative effects of complications of delivery management, poor social support, vaginal mode of delivery, anxiety, poor physical and mental quality of life on functional status of postpartum women. In addition, interventions targeting at mechanisms of improving social support could enhance the functional status of mothers in the postpartum period.

## Supplementary Information

**Additional file 1.**

**Additional file 2.**

## Data Availability

Extra data is available from the corresponding author upon reasonable request.

## Abbreviations

CFA: Confirmatory factor analysis
CFI: comparative fit index
DSM-5: Diagnostic and Statistical Manual of Mental Disorders version 5
HRQOL: Health related quality of life
OR: Odds ratio
PCL-5: Posttraumatic Stress Disorder Checklist version 5
PTSD: posttraumatic stress disorder
RMSEA: Root-mean-square approximation error
SEM: Structural equation modelling
TLI: Tucker-Lewis’s index
W-DEQ: Wijma Delivery Expectation/Experience Questionnaire
WHODAS 2.0: World Health Organization Disability Assessment Schedule version 2
WHO: World Health organization
