# Supplementary material for: A structural equation modelling of the direct and indirect factors associated with functional status over time as measured by WHODAS-32 items among postpartum women in Northwest Ethiopia
Source: Arch Public Health. 2023 Mar 18;81:41. doi: 10.1186/s13690-023-01055-w (PMC10024387; doi:10.1186/s13690-023-01055-w)
Supplement: Supplementary file 1 — Additional file 1. [file 13690_2023_1055_MOESM1_ESM.docx]

**Measurement tools used for the variables**

**Depression, anxiety and stress**

The abbreviated form of the Depression, Anxiety and Stress Scale-21 questionnaire was used to measure depression, anxiety, and stress[1]. With 21 items in three categories, it is a reliable and validated instrument. Each domain has seven items that measure stress, anxiety, and depression symptoms. In this study, a mother was considered to have a depressive symptom if she scored greater than or equal to 10. For the purposes of this study, anxiety symptoms were defined as greater than or equal to the cutoff score of 8. For this study, a score of greater than or equal to 15 was deemed to indicate stress symptoms. This instrument has already been validated and used previously in Ethiopia [2, 3].

**Posttraumatic stress disorder**

We have used the Posttraumatic Stress Disorder Checklist for DSM-5 (PCL-5) comprising the 20 PTSD symptoms (criterion B, C, D and E) to measure PTSD over the past month. The instrument contains 20 items, including three new PTSD symptoms (compared with the PTSD Checklist for DSM-V): blame, negative emotions and reckless or self-destructive behavior[4]. Summing the items gives a total-symptom score ranging from 0 to 80 [5, 6]. A value of ≥ 33 was regarded to have symptoms of PTSD for in this research. The cut-offs for the instrument were validated by a previous study in Ethiopia[6].

**Fear of childbirth**

Fear of childbirth was assessed using the Wijma Delivery Expectation/Experience Questionnaire (W-DEQ). The W-DEQ was designed specifically to assess fear of childbirth as operationalized by a cognitive evaluation of the delivery. The internal consistency and split-half reliability of the W-DEQ was checked in previous studies in Ethiopia with the Cronbach’s alpha score of 0.932[7, 8]. For this study, a score of ≥85 was used to have fear of childbirth [7, 8].

**Social support**

The Oslo three-item Social Support Scale, which has scores ranging from 3 to 14, was used to measure social support. For scores below 9, the social support was classified as poor or no social support. The results from 9 to 14 were combined to represent "yes" for social support because they were regarded as moderate to high support. Ethiopian researchers verified and previously employed the Oslo three-item Social Support Scale [9-11].

**References:**

1. Malaju MT, Alene GD, Azale T. Longitudinal functional status trajectories and its predictors among postpartum women with and without maternal morbidities in Northwest Ethiopia: a group based multi-trajectory modelling. BMJ Global Health. 2022;7(1):e007483.

2. Yeshaw Y, Mossie A. Depression, anxiety, stress, and their associated factors among Jimma University staff, Jimma, Southwest Ethiopia, 2016: a cross-sectional study. Neuropsychiatr Dis Treat. 2017;13:2803-12.

3. Abebe AM, Kebede YG, Mengistu F. Prevalence of Stress and Associated Factors among Regular Students at Debre Birhan Governmental and Nongovernmental Health Science Colleges North Showa Zone, Amhara Region, Ethiopia 2016. Psychiatry J. 2018;2018:7534937.

4. Weathers FW, Litz BT, Keane TM, Palmieri PA, Marx BP, Schnurr PP. The ptsd checklist for dsm-5 (pcl-5). Scale available from the National Center for PTSD at www ptsd va gov. 2013;10.

5. Verhey R, Chibanda D, Gibson L, Brakarsh J, Seedat S. Validation of the posttraumatic stress disorder checklist - 5 (PCL-5) in a primary care population with high HIV prevalence in Zimbabwe. BMC psychiatry. 2018;18(1):109-.

6. Smyth-Dent K, Fitzgerald J, Hagos Y. A Field Study on the EMDR Integrative Group Treatment Protocol for Ongoing Traumatic Stress Provided to Adolescent Eritrean Refugees Living in Ethiopia. Psychology and Behavioral Science International Journal. 2019;12(4):1-12.

7. Gelaw T, Ketema TG, Beyene K, Gurara MK, Ukke GG. Fear of childbirth among pregnant women attending antenatal care in Arba Minch town, southern Ethiopia: a cross-sectional study. BMC Pregnancy and Childbirth. 2020;20(1):1-7.

8. Yetwale A, Melkamu E. Fear of Childbirth and Associated Factors Among Pregnant Mothers Who Attend Antenatal Care Service at Jinka Public Health Facilities, Jinka Town, Southern Ethiopia. International Journal of Childbirth. 2021.

9. Bezabh YH, Abebe SM, Fanta T, Tadese A, Tulu M. Prevalence and associated factors of post-traumatic stress disorder among emergency responders of Addis Ababa Fire and Emergency Control and Prevention Service Authority, Ethiopia: institution-based, cross-sectional study. BMJ open. 2018;8(7):e020705.

10. Asnakew S, Shumet S, Ginbare W, Legas G, Haile K. Prevalence of post-traumatic stress disorder and associated factors among Koshe landslide survivors, Addis Ababa, Ethiopia: a community-based, cross-sectional study. BMJ open. 2019;9(6):e028550.

11. Denur M, Tesfaw G, Yohannis Z. The magnitude and correlates of common mental disorder among outpatient medical patients in Ethiopia: an institution based cross-sectional study. BMC research notes. 2019;12(1):360.
