# Supplementary material for: A structural equation modelling of the direct and indirect factors associated with functional status over time as measured by WHODAS-32 items among postpartum women in Northwest Ethiopia
Source: Arch Public Health. 2023 Mar 18;81:41. doi: 10.1186/s13690-023-01055-w (PMC10024387; doi:10.1186/s13690-023-01055-w)
Supplement: Supplementary file 2 — Additional file 2. [file 13690_2023_1055_MOESM2_ESM.docx]

**Model fitness criteria for the structural equation modelling**

We determined the model fitness using the comparative fit index (CFI), Tucker-Lewis’s index (TLI), root-mean-square approximation error (RMSEA) with a Sartorra-Bentler correction method. The TLI and CFI should both be greater than 0.90, and the RMSEA value should be less than 0.08, in order to assess whether the model is reasonably fitting the data [1-3]. It is recommended to use the Sartorra-Bentler adjustment approach when the multivariate normality assumption of structural equation modeling is violated[3, 4]. The chi-square test has also been advocated for use in evaluating the model's fitness, but it has drawn criticism for its sensitivity to sample size [3, 5, 6]. The chi-square test might reject promising models due to a high sample size or data that are not normally distributed because of its sensitivity to sample size, which increases type II error[3, 5]. This was accurate when applying the chi-square test to determine the model fitness of our study. With regard to other model fitness criteria, the model fitness for this study was good, but not with the chi-square test, which suggests that combining the chi-square test with a large sample size results in the rejection of a decent model. The likelihood of achieving a statistically significant chi-square increases with sample size[3, 7]. The chi-square test is assured to be significant, even at higher significance cutoffs, because SEM should only be performed with large sample sizes[3, 7]. Therefore, other measures of fit must be taken into account because the chi-square statistic is sensitive to sample size[3, 7]. Because of this, it seems that the best course of action is to employ a selection of fit indices that calculate exact model fit based on chi-square (standardized root mean square residual), relative fit indices that compare the hypothesized model to an independent baseline model (Tucker-Lewis index), and non-centrality-based indices that test the alternative hypothesis rather than the null (root mean square error of approximation)[3, 6].

**References:**

1. Ginzburg K, Ein-Dor T, Solomon Z. Comorbidity of posttraumatic stress disorder, anxiety and depression: a 20-year longitudinal study of war veterans. Journal of affective disorders. 2010;123(1-3):249-57.

2. Haagen JF, Moerbeek M, Olde E, Van Der Hart O, Kleber RJ. PTSD after childbirth: A predictive ethological model for symptom development. Journal of affective disorders. 2015;185:135-43.

3. Malaju MT, Alene GD, Bisetegn TA. Longitudinal path analysis for the directional association of depression, anxiety and posttraumatic stress disorder with their comorbidities and associated factors among postpartum women in Northwest Ethiopia: A cross-lagged autoregressive modelling study. PloS one. 2022;17(8):e0273176.

4. Xia Y, Yung Y-F, Zhang W. Evaluating the selection of normal-theory weight matrices in the Satorra–Bentler correction of chi-square and standard errors. Structural Equation Modeling: A Multidisciplinary Journal. 2016;23(4):585-94.

5. Gomer B, Jiang G, Yuan K-H. New effect size measures for structural equation modeling. Structural Equation Modeling: A Multidisciplinary Journal. 2019;26(3):371-89.

6. Perry JL, Nicholls AR, Clough PJ, Crust L. Assessing model fit: Caveats and recommendations for confirmatory factor analysis and exploratory structural equation modeling. Measurement in physical education and exercise science. 2015;19(1):12-21.

7. Schumacker E, Lomax G. A Beginner’s Guide to Structural Equation Modelling. 4th edtn. London: Routledge; 2016.
